# Extraction Kinetics of phytochemicals and antioxidant activity during black tea (*Camellia sinensis* L.) brewing

**DOI:** 10.1186/s12937-015-0060-x

**Published:** 2015-07-31

**Authors:** Chamira Dilanka Fernando, Preethi Soysa

**Affiliations:** 1 Department of Biochemistry & Molecular Biology, Faculty of Medicine, University of Colombo, Kynsey Road, Colombo 08, Sri Lanka; 2 Institute of Chemistry Ceylon, College of Chemical Sciences, Adamantane House, 341/22, Kotte Road, Welikada, Rajagiriya, Sri Lanka

**Keywords:** *Camellia sinensis* L, Black tea, Brewing time, Extraction kinetics

## Abstract

**Background:**

Tea is the most consumed beverage in the world which is second only to water. Tea contains a broad spectrum of active ingredients which are responsible for its health benefits. The composition of constituents extracted to the tea brew depends on the method of preparation for its consumption. The objective of this study was to investigate the extraction kinetics of phenolic compounds, gallic acid, caffeine and catechins and the variation of antioxidant activity with time after tea brew is made.

**Methods:**

CTC (Crush, Tear, Curl) tea manufactured in Sri Lanka was used in this study. Tea brew was prepared according to the traditional method by adding boiling water to tea leaves. The samples were collected at different time intervals. Total phenolic and flavonoid contents were determined using Folin ciocalteu and aluminium chloride methods respectively. Gallic acid, caffeine, epicatechin, epigallocatechin gallate were quantified by HPLC/UV method. Antioxidant activity was evaluated by DPPH radical scavenging and Ferric Reducing Antioxidant Power (FRAP) assays.

**Results:**

Gallic acid, caffeine and catechins were extracted within a very short period. The maximum extractable polyphenols and flavanoids were achieved at 6–8 min after the tea brew is prepared. Polyphenols, flavanoids and epigallocatechin gallate showed a significant correlation (*p* < 0.001) with the antioxidant activity of tea.

**Conclusion:**

The optimum time needed to release tea constituents from CTC tea leaves is 2–8 min after tea is made.

## Background

Oxidation is an essential biological process for energy production reactions in living organisms. However, excessive *in vivo* production of reactive oxygen species (ROS) leads to oxidative damage in lipids, protein and DNA. Oxidative damages cause the risk of development of neuro degeneration, mutagenesis, carcinogenesis, coronary heart disease, diabetes and cellular ageing [1]. Recently an increasing number of scientific publications suggest that certain edible plants may offer protection or treatment against some chronic diseases. In general, these beneficial effects can be attributed to their antioxidant constituents such as vitamin C, vitamin E, carotenoids, flavonoids, catechins, anthocyanins, etc. [2].

Tea is the most consumed beverage in the world only second to water. CTC (Crush, Tear, Curl) tea is manufactured in Sri Lanka from the two leaves and the bud and young leaves of the shrub *Camellia sinensis* (L.) Kuntze (family: Theaceae) [3]. The estimated amount of 18–20 billion tea cups consumed daily worldwide elicits its economic and social interest [4]. Most of the tea produced worldwide is black tea, which represents 76–78 % of the tea produced and consumed worldwide [5]. The production of black tea involves allowing tea leaves to wither where the moisture content of the leaves is reduced followed by rolling and crushing to initiate fermentation [6].

The chemical composition of tea includes polyphenols, alkaloids (caffeine and theobromine), amino acids (mainly L-theanine), carbohydrates, proteins, chlorophyll, volatile compounds, minerals (aluminium, manganese and fluoride) and other unidentified compounds. Among these, polyphenols are one of the main bioactive compounds in tea [7]. The major polyphenolic compounds in tea are the flavan-3-ols (catechins) which include: (−)-epicatechin (EC), (−)-epigallocatechin (EGC), (−)-epicatechin gallate (ECG), (−)-epigallocatechingallate (EGCG), (−)-gallocatechins (GC) and (−)-gallocatechin gallate (GCG) [8]. During the manufacturing process of black tea, the colourless catechins present in fresh tea leaves are oxidized both enzymatically and nonenzymatically to give two major groups of pigments: theaflavins and thearubigins which are responsible for the colour and sensory properties of black tea brew [9]. The flavonoids and other polyphenols present in tea have shown a wide range of biological and pharmaceutical benefits, including prevention of cancer [10], obesity [11], type 2 diabetes [12], depressive symptoms [13] cardiovascular diseases and cerebral ischemic damage [14]. Further tea possesses insulin-enhancing [15], antioxidative [16], hypolipidemic [17], antimicrobial [18], immune-stimulatory [19], anti-inflammatory [20], neuroprotective [21] and bone mineralization enhancement activities [22]. These beneficial effects may be attributed to antioxidant activity possessed by the polyphenolic compounds in tea [7]. Gallic acid has proven its cytotoxic activity against cancer cell lines [23]. Epidemiological studies have found a positive association between dietary flavonoid intake and overall good health [24].

Stirring and steeping conditions such as time and temperature are critical factors for extraction of catechins or theaflavins from teas [25]. The traditional method of making a cup of tea in some countries including Sri Lanka is to place loose leaves directly into a pot and pour boiling water over the leaves. After a couple of minutes the leaves are usually removed and the infusion is consumed. The extractable phytochemicals associate with the health benefits and their levels depend on the method of tea preparation for its consumption. Therefore, this study aims to evaluate the kinetics of solid liquid extraction of polyphenols, flavonoids, gallic acid, caffeine, epicatechin and epigallocatechin gallate in tea infusion made with CTC tea.

## Methods

### Reagents and chemicals

HPLC grade acetonitrile was purchased from BDH (BDH Chemicals Ltd. Poole, England). β-hydroxyethyltheophylline, caffeine, gallic acid, epicatechin, (−)-epigallocatechin gallate, Folin Ciocalteu reagent, 1,1-Diphenyl-2-picrylhydrazyl (DPPH) and aluminium chloride were purchased from Sigma Chemicals, USA. Sodium nitrite was purchased from Riedel De Haen Ag, Wunstorfer Strasse 40, SEELZE1, D3016, Germany. Crush, Tear, Curl (CTC) low grown pure Ceylon black tea was obtained from Danduwangalawatta Tea factory, Millawitiya, Kuruwita, Sri Lanka.

### Equipment

Shimadzu UV-1601 UV Visible spectrophotometer (Shimadzu Corporation, Japan) was used to read the absorbance. HPLC was performed with Shimadzu LC 10AS solvent delivery system equipped with UV/VIS variable wavelength detector Shimadzu SPD 10A (Shimadzu Corporation, Japan) and an integrator Shimadzu C-R8A (Shimadzu Corporation, Japan). Chromatographic resolution of components in tea was achieved on betasil phenyl HPLC column (2.1 x 150 mm) from Thermo scientific. Samples were injected with a syringe loading injector fitted with a 100 μl loop.

Shimadzu Libror AEG-220 analytical balance (Shimadzu Corporation, Japan) was used to prepare standard solutions. Purified deionized water was obtained from Labconco Water Pro-PS UV ultra filtered water system (Labconco Corporation, Missouri) and distilled water was obtained by Aquatron A4S water system. Micro-centrifugation was performed using a BioFuge-Pico D-37520 centrifuge (Heraeus Instruments, Germany).

### Preparation of tea brew

Tea brew was prepared according to the conventional method. Deonized water (500 ml) was boiled in a glass beaker placed on a hot plate. At the onset of boiling, heating was terminated and the tea leaves (5.0 g) were added to boiled water. The beaker was then covered with a watch glass. Magnetic stirrer was used at a constant speed to maintain a homogenous sample. A volume of 1.0 ml was withdrawn at different time intervals (0, 1, 2, 4, 6, 8, 10, 12, 14, 20 min) and centrifuged. The supernatant was assayed for their phenolic and flavonoid content by spectrophotometry. Gallic acid, caffeine, epicatechin and epigallocatechin gallate were quantified by Reversed Phase High Pressure Liquid Chromatography (RP-HPLC). Antioxidant activity was assayed by DPPH radical scavenging and Ferric reducing Antioxidant Power (FRAP) methods.

### Determination of phenolic content

Total phenolic content was determined by Folin Ciocalteu method [26]. Samples (25 μl) were diluted up to 1500 μl with deionized water. Folin Ciocalteu’s reagent (1 N, 250 μl) was added to the samples (500 μl), and the mixture was allowed to stand at room temperature for 2 min. Sodium carbonate solution (10 %, 1.25 ml) was then added and incubated for 45 min in the dark at room temperature. The absorbance of the resulting solution was measured at 760 nm against a blank prepared with deionized water. Calibration curves were constructed with gallic acid and (−)-epigallocatechin gallate (EGCG) standards. The total phenolic content was expressed as gallic acid equivalents (GAE) mg/g of tea leaves as well as EGCG equivalents mg/g of tea leaves. Tea samples brewed independently were analyzed in replicates (*n* = 6).

### Determination of flavonoid content

The flavonoid content was measured by the aluminum chloride colorimetric assay [27]. Tea brew (25 μl) collected at different time intervals were diluted with deionized water up to 500 μl and mixed with sodium nitrite (5 %, 30 μl). After 5 min aluminium chloride (10 %, 30 μl) was added to the mixture followed by sodium hydroxide (1 M, 200 μl) at the 6^th^ minute. The final volume was adjusted to 1000 μl with deionized water and absorbance was measured at 510 nm against a blank prepared with deionized water replacing the tea brew. Calibration curve was plotted using EGCG standards and flavonoid content was expressed as EGCG equivalents mg/g of tea leaves. Tea brew prepared independently were analyzed in replicates (*n* = 6).

### Antioxidant capacity by 1,1-Diphenyl-2-picrylhydrazyl (DPPH) radical assay

Free radical scavenging ability of tea samples collected at different time intervals and authentic samples of tea constituents (gallic acid, caffeine, epicatechin and epigallocatechin gallate) was assayed by DPPH radical scavenging method with slight modifications [28]. Test samples (50 μl) were diluted up to 1000 μl with deionized water. DPPH reagent prepared in absolute ethanol (100 μM, 950 μl) was added to the test sample (50 μl) and the mixture was allowed to stand for 30 min in the dark. The scavenging activity was quantified by measuring the absorbance at 517 nm. Deionized water was used as the blank. The control was prepared by mixing deionized water (50 μl) with DPPH (950 μl). Results were expressed as percentage scavenging of DPPH radical calculated using the following equation:

$$ \%\ \mathrm{Scavenging}\ \mathrm{of}\ \mathrm{DPPH}\ \mathrm{free}\ \mathrm{radical}=\frac{\mathrm{Abs}.\ \mathrm{of}\ \mathrm{control}-\mathrm{Abs}.\ \mathrm{of}\ \mathrm{sample}}{\mathrm{Abs}.\ \mathrm{of}\ \mathrm{control}} \times 100\% $$

Percentage scavenging of DPPH radical against time was plotted. Tea brew prepared independently was analyzed in replicates (*n* = 6) for antioxidant activity. L-Ascorbic acid was used as the standard antioxidant. The effective concentration needed to scavenge 50 % of the DPPH radical with respect to the control (EC_50_) was calculated for each of the tea constituents and the standard antioxidant.

### Antioxidant capacity by ferric reducing antioxidant power (FRAP) assay

The ferric ion reducing power of the samples collected at different time intervals was determined according to Sharma and Kumar (2011) with slight modifications [29]. Samples (50 μl) were diluted up to 1000 μl with deionized water. The test sample (100 μl) was mixed with phosphate buffer (0.2 M, pH 6.6, 250 μl) and potassium ferricyanide (1 %, 250 μl). The mixture was incubated at 50 °C for 20 min. Trichloroacetic acid (10 %, 250 μl) was added and the samples were centrifuged at 6500 rpm for 10 min. The supernatant was mixed with deionized water and ferric chloride (0.1 %) at a ratio of 1:1:2 respectively. The samples were vortexed and absorbance was measured at 700 nm. The reagent blank was prepared by replacing tea sample with deionized water. L-ascorbic acid was used as the standard antioxidant. The antioxidant capacity was expressed as Ascorbic acid equivalent reducing power (mg/g of tea leaves).

### Determination of Gallic acid, Caffeine, Epicatechin (EC) and (−)-Epigallocatechin gallate (EGCG) using Reversed Phase High Pressure Liquid Chromatography

Tea brew was diluted as necessary for the quantification of gallic acid, epicatechin and (−)-epigallocatechin gallate and caffeine. Samples (100 μl) were mixed with the internal standard, β–hydroxyethyltheophilline (10 μg/ml, 100 μl) and centrifuged (2000 rpm, 5 min). The supernatant (25 μl) was injected onto the HPLC column. The mobile phase used was isocratic elution system consists of 8 % acetonitrile, 1 % glacial acetic acid and 91 % deionized water at a flow rate of 0.5 ml/min. The peaks were detected at 280 nm. Standards were prepared with a mixture of gallic acid, caffeine, EC and EGCG (2.5 – 25 μg/ml) in deionized water. Calibration curve was constructed using peak area ratio of gallic acid, caffeine, EC and EGCG (ratio of peak area of the relevant standard to that of the internal standard) against the concentration. The HPLC method was validated for accuracy, intraday and interday precision, linearity, limit of detection (LOD) and limit of quantitation (LOQ) according to the guidelines provided [30].

### Evaluation of kinetics of releasing phytochemicals from tea leaves

Kinetics of solid liquid extraction of polyphenols, flavanoids, gallic acid, caffeine, EC and EGCG mixtures were studied.

Second-order rate law for extraction of compounds from tea leaves is considered [31];

1 $$ \frac{dc}{dt}={k}_1{\left(c-{c}_{\infty}\right)}^2 $$

k_1_ = the second-order extraction rate constant (g μg^−1^ min^−1^)

C_∞_ = the extraction capacity (concentration of tea constituents at saturation in g L^−1^)

C = the concentration of tea constituents in the solution at any time (g L^−1^), t (min)

By considering the boundary condition t = 0 to t and C = 0 to C, the integrated rate law for a second-order extraction was obtained.

2 $$ c=\frac{c_{\infty}^2{k}_1t}{1+{c}_{\infty }{k}_1t} $$

By linear transformation of the above equation, the rate constant *k*_*1*_ can be determined by fitting the experimental data.

3 $$ \frac{t}{c}=\frac{1}{k_1{c}_{\infty}^2}+\frac{t}{c_{\infty }} $$

### Statistical analysis

Results are presented as mean ± standard deviation (Mean ± SD) of six independent experiments. Statistical analysis, student’s *t*-test and calculation of Pearson’s correlation coefficient (*r*) were performed using Microsoft Excel. Value of *p* < 0.05 was considered as significant.

## Results and discussion

Gallic acid, caffeine, EC, EGCG and the internal standard β-hydroxyethyltheophilline were eluted at 1.96, 5.89, 12.21, 15.49 and 4.21 min respectively. The calibration curves were linear over 2.5 – 25 μg/ml with R^2^ exceeding 0.995 for gallic acid, caffeine, EC and EGCG. The method showed repeatability, interday and intraday precision (CV %) less than 6.0 % and accuracy was 96–103 % for all the compounds studied.

**Fig. 1:**
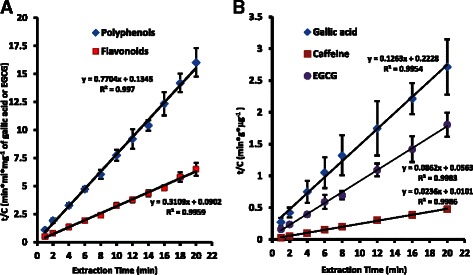
Correlation between t/C and extraction time for polyphenols and flavonoids (**a**) and correlation between t/C and extraction time for gallic acid, caffeine and epigallocatechin gallate (**b**)

Concentrations obtained for all the compounds tested were fitted with the second order kinetics. Linear curves (R^2^ > 0.99) were obtained for time against time/concentration (t/C) as described by many authors [32, 33] for all individual set of data (*n* = 6) for solid liquid extraction of each compound investigated. Curves obtained from the mean values of each compounds are illustrated in the Fig. 1. The gradient and intercept values of the curves were used to determine the extraction capacity (C_∞_) and second-order extraction rate constant (*k*_*1*_). These kinetic parameters were calculated from the data obtained during first 5 min of tea brewing, assuming the change of temperature to be negligible during this period. However the effect of temperature has not affected the linearity of the curves (Fig. 1). The calculated values with the observed values are listed in Table 1.

**Table 1:** Kinetic parameters for the extraction of nutraceuticals from tea infusion

	C_∞_ (mg/g)	C_20_ (mg/g) (observed)	*k*_*1*_ (g mg^−1^ min^−1^)
GA	8.0 ± 1.2	7.6 ± 1.9	7.8 x10^−2^ ± 0.2 x10^−2^
Caffeine	42.2 ± 2.7	42.1 ± 1.9	4.1 x10^−3^ ± 0.3 x10^−3^
EGCG	11.8 ± 0.4	11.2 ± 1.1	1.4 x10^−2^ ± 0.8 x10^−2^
Polyphenols	131 ± 8^a^	124 ± 6^a^	2.4 x10^−2^ ± 0.7 x10^−2^
Flavanoids	322 ± 22^b^	310 ± 26^b^	5.8 x10^−4^ ± 0.1x10^−4^

^a^Concentration is expressed in mg/g GAE and ^b^Concentration is expressed in mg/g EGCG equivalents

(C_∞_ = the extraction capacity, C_20_ = the concentration of the tea constituent in the solution at 20^th^ minute, *k*_*1*_ = the second-order extraction rate constant)

**Fig. 2:**
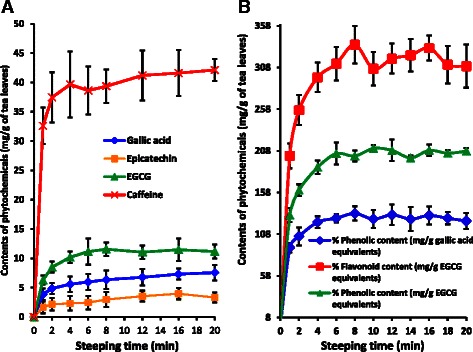
Kinetics of caffeine, gallic acid and catechin extraction from CTC tea leaves (**a**) and kinetics of polyphenol and flavonoid extraction from CTC tea leaves (**b**)

Rapid extraction was observed for gallic acid, caffeine, EC, EGCG within first two minutes and slowly increased with time (Fig. 2a). The release of compounds from tea leaves depends on the amount present and their solubility. All the compounds investigated in the present study are water soluble. Caffeine is soluble in polar and non polar solvents and efficient release can be expected as observed in the present study. Furthermore caffeine present in the tea leaf is higher compared to the other three compounds investigated. The release of total phenols and flavonoids also showed rapid and slow release phases but comparatively slower than caffeine, gallic acid and other two catechins (Fig. 2a and b). Total polyphenolic and flavonoid compounds extracted increased with the time and reached to a maximum concentration at 6–8 min (Fig. 2b). The values which represent total polyphenolic content (TPC) and flavonoid content account for all the polyphenolic substances present in tea leaves having different solubility which could be responsible for slow release of TPC with time. The maximum concentrations observed for total phenols and flavonoids were 13.28 ± 0.86 w/w % GAE and 33.53 ± 2.26 w/w % EGCG equivalents respectively. At low temperature caffeine complexes with polyphenols and forms insoluble complexes [34]. It is observed that the temperature decreased from 100–75 °C during the first twenty minutes, but complexation of polyphenols with caffeine was not visible.

**Fig. 3:**
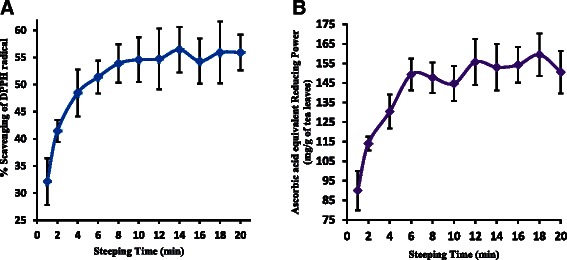
Variation of % scavenging of DPPH free radical with time (**a**) and variation of Ascorbic acid equivalent Ferric Reducing Antioxidant Power with time (**b**)

**Fig. 4:**
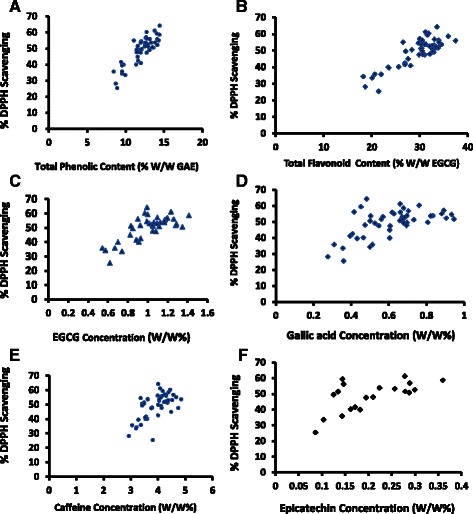
Correlation of antioxidant activity with the concentration of polyphenols (**a**), flavonoids (**b**), EGCG (**c**), gallic acid (**d**), caffeine (**e**) and EC (**f**)

Most of the health benefits of tea are based on its antioxidant activity. According to DPPH and FRAP assays, the antioxidant activity was increased with the steeping time (Fig. 3a and b). A significant correlation (*p* < 0.001) was observed between the antioxidant activity with polyphenols, flavonoids and EGCG (Fig. 4a – f). A moderate or low correlation was observed between antioxidant activity and gallic acid, EC and caffeine present in the tea brew. Though the antioxidant capacity of gallic acid and EC is high (Table 2), their contribution in tea brew to scavenge DPPH is low since their concentration is relatively low when compared with EGCG and phenols. Present study reveals that polyphenols and EGCG are major constituents responsible for antioxidant activity. Although caffeine is not considered as a polyphenolic compound, its capability to inhibit lipid peroxidation has been reported [35]. However the authors suggest that its contribution to antioxidant activity in coffee and tea does not seem to be as important as the contribution of phenolic compounds. This is in agreement with the results obtained in the present study.

**Table 2:** EC_50_ values obtained in the DPPH assay for authentic samples of tea constituents and the standard antioxidant

Compound	EC_50_ for DPPH Assay
Gallic acid	1.38 ± 0.04 μg/ml
Epicatechin	2.30 ± 0.06 μg/ml
Epigallocatechin gallate	3.24 ± 0.07 μg/ml
L-Ascorbic acid	3.30 ± 0.27 μg/ml
Caffeine	>1.25 mg/ml

## Conclusions

The traditional method of preparing a CTC tea brew has influence on the extraction of total phenols, flavonoids and major phytoconstituents such as gallic acid, caffeine, EC and EGCG. The antioxidant capacity of CTC black tea significantly correlated well with levels of EGCG, flavonoids and total phenolics during steeping of the black tea. A very weak correlation was observed between caffeine content with antioxidant activity. Releasing of caffeine, gallic acid, EGCG and EC into the tea brew was much faster than the release of polyphenols and flavonoids. The minimum brewing time required to release tea constituents from CTC tea leaves is 2–8 min after tea is prepared according to the traditional method.

Since tea is one of the major beverages consumed worldwide, studying the effect of preparation of tea on the extractability of various health beneficial phytoconstituents is highly valued.
